# Outcomes of an Accelerated Inpatient Refeeding Protocol in 103 Extremely Underweight Adults with Anorexia Nervosa at a Specialized Clinic in Prien, Germany

**DOI:** 10.3390/jcm9051535

**Published:** 2020-05-19

**Authors:** Thorsten Koerner, Verena Haas, Julia Heese, Matislava Karacic, Elmar Ngo, Christoph U. Correll, Ulrich Voderholzer, Ulrich Cuntz

**Affiliations:** 1 Schön Clinic Roseneck; Am Roseneck 6, 83209 Prien am Chiemsee, Germany; thkoerner@schoen-klinik.de (T.K.); jheese@schoen-klinik.de (J.H.); mkaracic@schoen-klinik.de (M.K.); engo@schoen-klinik.de (E.N.); UCuntz@schoen-klinik.de (U.C.); 2 Clinic for Psychiatry, Psychosomatics and Psychotherapy of childhood and adolescence, Charité – University Berlin, corporate member of Free University Berlin, Humboldt-University Berlin, and Berlin Institute of Health, 13353 Berlin, Germany; verena.haas@charite.de (V.H.); christoph.correll@charite.de (C.U.C.); 3 Department of Psychiatry and Molecular medicine, Donald and Barbara Zucker School of Medicine at Hofstra/Northwell, Hempstead, NY 11549, USA; 4 Department of Psychiatry, the Zucker Hillside Hospital, Glen Oaks, NY 11004, USA; 5 Department of Psychiatry and Psychotherapy, Ludwig-Maximilians-Universität München (LMU), 80336 München, Germany; 6 Research program for the evaluation of psychotherapy in complex therapeutic settings, PMU Paracelsus Medical University Salzburg, 5020 Salzburg, Austria

**Keywords:** anorexia nervosa, caloric intake, refeeding syndrome, refeeding protocol

## Abstract

Background: In mildly to moderately malnourished adolescent patients with anorexia nervosa (AN), accelerated refeeding protocols using higher initial calory supply coupled with phosphate supplements were not associated with a higher incidence of refeeding syndrome (RS). It is unclear whether this is also a feasible approach for extremely malnourished, adult AN patients. Methods: Outcomes of a clinical refeeding protocol involving a targeted initial intake of ≥2000 kcal/day, routine supplementation of phosphate and thiamine as well as close medical monitoring, were evaluated. A retrospective chart review including AN patients with a body mass index (BMI) <13 kg/m² was conducted, to describe changes in weight, BMI, and laboratory parameters (phosphate, creatine kinase, hematocrit, sodium, liver enzymes, and blood count) over four weeks. Results: In 103 female patients (age, mean ± standard deviation (SD) = 23.8 ± 5.3 years), BMI between admission and follow-up increased from 11.5 ± 0.9 to 13.1 ± 1.1 kg/m² and total weight gain within the first four weeks was 4.2 ± 2.0 kg (mean, SD). Laboratory parameter monitoring indicated no case of RS, but continuous normalization of blood parameters. Conclusions: Combined with close medical monitoring and electrolyte supplementation, accelerated refeeding may also be applied to achieve medical stabilization in extremely underweight adults with AN without increasing the risk of RS.

## 1. Introduction: Refeeding and Refeeding Syndrome

Refeeding syndrome (RS) is a sudden, threatening deterioration in the general physical condition of a cachectic patient undergoing refeeding, especially in the first two to three weeks of refeeding following a prolonged period of hunger. RS has been scientifically described, among other conditions, in prisoners of war after the Second World War [1,2] and later in the refeeding of predominantly older underweight patients and patients with tumor cachexia [3,4]. Characteristic features of RS are fluid shifts and electrolyte fluctuations, which can occur as a result of endocrine and metabolic changes resulting from the onset of refeeding. At the center of RS are hypophosphatemia, hyponatremia, disturbed glucose metabolism, and insufficient thiamine supply [5]. Drastic malnutrition is associated with depleted intracellular phosphate stores. Initially, refeeding causes a change from a catabolic to anabolic metabolism, and concomitant hyperparathyroidism may contribute to the development of hypophosphatemia [6]. This process can lead to a critical drop in the intracellular concentration of adenosine triphosphate (ATP) and thus in the energy supply of the cells [7]. Clinical consequences range from severe organ dysfunction and rhabdomyolysis to seizures, delirium, coma, and death. The definition of RS is not clear-cut, and so there are no clear prevalence figures for RS in patients with anorexia nervosa (AN). In adult AN patients, the incidence of hypophosphatemia during refeeding is reported to range from 0%–80% [8,9]. Appropriate control of energy intake, as well as monitoring and adequate supplementation of electrolytes, are essential in the early stages of refeeding to avoid complications [10].

Due to the fear of serious medical complications related to RS, “start low, go slow” approaches (e.g., starting at 1200 kcal/d and increasing by 200 calories every other day) have been practiced for a long time in the refeeding of patients with AN. In practice, this approach leads initially to a hypocaloric diet and the maintenance of severe energy deficiency and to additional weight loss, which, in some cases, can lead to pronounced hypoglycemia with subsequent organ damage, as well as to longer hospital stays. In adolescent patients with AN, higher initial weight gain has been linked with a higher likelihood of remission by the end of treatment [11].

There is currently no consensus regarding the adequate initial energy supply during the refeeding of AN patients, which leads to inconsistent procedures. A scientific basis for the very restrictive dietary guidelines for the initial caloric intake in severely underweight adult patients is still missing, and presumably, the initial BMI [12] or the expression of cachexia is a predictor of RS [13]. Study protocols, in which an initially higher caloric intake was compared with a low-caloric diet [14] or with high energy intake in the first week and a gradual reduction of energy supply [15], showed no difference in the occurrence of components of RS, but larger and faster weight gain with the higher-caloric refeeding approach. In addition, a review of 22 studies by Garber et al. [16] showed that in mildly to moderately malnourished AN patients a low initial energy intake was too conservative and that higher initial energy levels were not associated with an increased risk of RS, as long as electrolytes, water balance, and cardiovascular parameters were closely monitored and controlled; however, most studies included in the review were conducted in adolescents. For extremely underweight adult patients, there are currently only a few studies examining higher caloric refeeding, and therefore no commonly accepted, reliable recommendations regarding the value and safety of accelerated refeeding strategies exist. In a recent study on 119 adults with AN, higher initial caloric refeeding (i.e., 1500 instead of 1000 kcal/d) was described to provide additional nourishment, to medically stabilize patients, and to prevent RS [17]. The authors concluded that future research is needed to examine whether higher-calorie intakes (i.e., 2000 kcal/d), similar to those studied in adolescent patients, may also be beneficial to treat adult patients.

This retrospective study aimed to describe (i) a clinical, high-caloric refeeding protocol established in a specialist ward of a psychosomatic clinic, targeting an initial intake of 2000 kcal/d and the accompanying essential medical measures; (ii) the outcomes of this approach regarding changes in weight and selected blood parameters to reflect physiological status and risk of RS.

## 2. Methods

### 2.1. Design and Population

The 24-bed ward featured in this study admitted patients with a diagnosis of anorexia nervosa who are at least 18 years old and who, due to their underweight, excessive exercising and/or vomiting and/or medical complications, require intensive medical and therapeutic surveillance.

For this retrospective chart review, patient data from all consecutive admissions were included if they fulfilled the following inclusion criteria:

- Age > 18 years; no upper age limit was defined as an inclusion criterion.
- Treated at a 24-bed unit for extremely underweight patients with 6 surveillance beds at Schön Klinik Roseneck, Rosenheim, Germany, between 1/1/2016 and 12/31/2018 (oral nutrition, no forced feeding or medication, no tube feeding, and all meals accompanied).
- Main diagnosis of AN (restrictive, active, and atypical AN).
- BMI ≤ 13 kg/m².
- Retention period of ≥28 days, as RS and related phenomena, are expected in this time window.
- Availability of laboratory data: five measurements from the time of admission until day 28.

Exclusion criteria were age <18 years, treatment at the institution outside of the time window, a BMI > 13 kg/m², and other eating disorder diagnoses, such as bulimia nervosa and binge eating disorders.

### 2.2. Ethical Approval

All procedures performed in studies involving human participants were in accordance with the ethical standards of the institutional review board of the Ludwig Maximilian Universität (LMU) Munich and with the 1964 Helsinki declaration and its later amendments or comparable ethical standards. According to the guidelines by the institutional review board of the LMU Munich, retrospective studies conducted on already available, anonymized data are exempt from requiring ethics approval.

### 2.3. Routine, Clinical Management of Meals, and Refeeding

All patients received three meals with an average total energy content of 2000 kcal per day from day 1 after admission to the ward, divided into three main meals with a choice between vegetarian and non-vegetarian menus. The caloric intake was adjusted and increased according to weight development to aim for an increase in body weight of 700–1000 g/week. The criterion for sufficient food intake was weight gain, which should be at least 100 g per day. If the agreed amount of food intake for weight gain could not be achieved, the portion size of one or more main meals was increased, and up to three snacks between meals were added. In addition, liquid food was offered to substitute for energy losses in case a part of the meals could not be eaten. All meals were therapeutically accompanied by a nurse or therapist in a 1:6 group supervision. Patient adherence to dietary intake was supported through daily therapeutic contacts and medical rounds. Patients ate their meals in a stable group setting and supported each other. Peer pressure may play an important role. In weekly eating protocol sessions, patients reviewed their progress and committed to new goals related to food avoidance, fears, and counteractive behavior. Patients did not receive nasogastric feeding since the normalization of eating behavior is a common therapeutic goal. All patients were prescribed 2000 kcal/d (three meals) starting from the day of admission. The average caloric value (data provided by the caterer and controlled in samples) for the non-vegetarian menu was 743, 717, and 704 (total 2164) kcal for breakfast, lunch, and dinner; and 743, 737, and 683 (total: 2162) for the vegetarian option. If patients did not finish their meal (50%–99% eaten) they were asked to drink 1 supplemental drink (400 kcal, Fresubin 200 mL with 2 kcal/mL). If patients ate less than 50% of their respective meal, they were asked to replace the missed-out calories by drinking 2 supplemental drinks (2 × 400 kcal). The group setting and the support from experienced therapists were considered essential for compliance with dietary adherence. Contingency measures were often necessary to regulate excessive exercising and slow weight gain. During the first 28 days, it was rare that patients required more than 2000 kcal/d but many patients received liquid food to replace unfinished meals. Patients reduced their calorie consumption by limiting their physical activity. Contingency contracts and video surveillance with 24/7 nursing staff presence as well as very moderate exercise therapy play an important role in normalizing movement behavior.

During the first 4 weeks, which was the observation period in this study, most patients gained sufficient weight with 2000 kcal/d spread over three meals. This was supported by restricting their physical activity and exercising. In individual cases, an energy intake of more than 4000 kcal was necessary to ensure sufficient weight gain. All patients participated in an intensive therapeutic program adapted to the special needs of the severely underweight women and suitable for therapeutically addressing the considerable anxieties and resistance associated with weight gain. This included daily individual cognitive behavioral therapy (CBT) sessions and medical rounds, accompanied meals, participation in group therapy (twice weekly problem-solving group, Cognitive Remediation Therapy (CRT), and art therapy, and daily morning and evening rounds with the nursing staff).

Daily documentation occurred for clinical and therapeutic observations and nursing contacts as well as weight measurement and comments on the accompanied meals. Physical examination including neurological assessment and vital signs was a standard procedure at admission and brief daily medical check during rounds. The SUSS (Sit up, squat, stand) test, as specified by the British Royal College of Psychiatrists MARSIPAN (Management of Really Sick Patients with AN) workgroup, assessed physical performance. It was used at admission and during rounds to follow up on physical performance. Patients used wheelchairs during the first weeks on the ward if their weight is below 13 kg/m². A normal result of the SUSS test plus a BMI of 13 kg/m² and above show that a wheelchair was no longer necessary. 

As part of the routine refeeding protocol: weight was monitored daily in underwear (Scale: Seca Model Nr. 6357021004). Height was measured at admission. Blood work was performed at least once a week including blood count (without white blood cell differential), electrolytes (sodium, potassium, phosphate, and chloride), transaminases, gamma-glutamyl-transferase (γ-GT), creatine kinase, creatinine, and urea. Further investigations included continuous monitoring of vital signs (electrocardiogram (ECG), heart rate, and blood pressure), hematocrit (<25%) and weight progression, routine clinical examination including neurological assessment and vital signs, SUSS test, sonography, and bioelectrical impedance analysis to assess edema status. Phosphate (612 mg to 1224 mg/d) and thiamine (200 mg/d) were supplemented routinely for two weeks and phosphate thereafter if needed. In some patients, the administration of diuretics was necessary at times when either the severity of the edema was subjectively too stressful or the heart rate per minute exceeded the systolic blood pressure in mmHg.

### 2.4. Data Assessment

The American Society of Parental and Enteral Nutrition developed consensus guidelines for the following diagnostic criteria for RS [18]: (i) a decrease of serum phosphorus, potassium, and/or magnesium levels by 10%–20% (mild RS), 20%–30% (moderate RS), or >30% and/or organ dysfunction resulting from a decrease in any of these and/or due to thiamin deficiency (severe RS) (ii) and occurring within 5 days of reinitiating or substantially increasing energy provision. These ASPEN criteria did not appear suitable to us for assessing the risk of the patients we treat in our unit: phosphate and thiamine were routinely supplemented and it was therefore unlikely that such a deficiency will occur in the context of malnutrition. Potassium was controlled even more closely (possibly several times a day), because of the risk of cardiac arrhythmia. If a drop in serum potassium occurred, potassium, as well as magnesium, were supplemented. In this respect, we chose not to adhere to these guidelines, and to report on a broad range of blood parameters to cover as many of the risks associated with re-nutrition as it was possible using our retrospective data from routine treatment.

The following criteria were used to define a case of RS:

- Critical deterioration of the general condition (e.g., severe edema, pericardial effusion, and weakness).
- Critical drop of serum phosphate to values <0.75 mmol/L.
- Increase in creatine kinase to >1000 U/L.
- Decrease of hematocrit to <25%.
- Decrease of serum sodium to <125 mmol/L.
- For the assessment of the clinical condition, the following laboratory parameters were also used:
  - Aspartate aminotransferase (GOT) (>35 U/L) and alanine aminotransferase (GPT) (>35 U/L) for estimation of liver cell damage.
  - Leukocytes (<3.98 G/L), hemoglobin (<11.2 g/L), and thrombocytes (<182 G/L) for the assessment of bone marrow function.

The following data were collected on admission and weekly thereafter for 4 weeks in clinical care and are presented in this study: body weight, height, BMI, and blood work (creatin kinase, phosphate, sodium, GOT, GPT, leucocytes, hematocrit, hemoglobin, and thrombocytes).

The following cut-off values were used to define pathological blood values: creatin kinase, >170 U/L; phosphate, <0.75 mmol/L; sodium, <12 mmol/L; GOT, GPT >35 U/L; leucocytes, <3.98 G/L, hemoglobin, <25%; thrombocytes, <182 G/L.

### 2.5. Statistical Analysis

All data were examined with the Shapiro–Wilk test for normal distribution and presented as mean ± standard deviation (normally distributed data) or as median and 25th/75th percentile (non-normally distributed data). For the change of BMI, weight, laboratory values over time, a paired samples t-test (for normally distributed differences), or a non-parametric, related samples Wilcoxon test (difference not normally distributed) was chosen. To assess how pathological laboratory values developed over time, the change in the percentage of pathological values in the total sample was treated as a categorical variable (pathological: y/n) and examined at the beginning and after 4 weeks using the two-sided Fisher’s Exact Test (https://www.graphpad.com/quickcalcs/contingency1.cfm). The Fisher’s Exact Test (Fisher–Yates test, exact chi-square test) is a significant test for independence in contingency tables. In contrast to the Chi-square independence test, however, it does not impose any requirements on the sample size and provides reliable results even with a small number of observations per cell.

A two-sided *p*-value <0.05 was considered statistically significant. Statistical analyses were performed by using SPSS version 21.0 for Windows (IBM Corp., Armonk, NY, USA).

## 3. Results

Between 01/01/2016 and 12/31/2018, 335 patients were admitted to the 24-bed unit for extremely underweight patients at Schön Klinik Roseneck in Rosenheim, Germany within a 120-bed hospital for psychosomatic medicine and psychotherapy. Of these 335 patients, 181 had a main diagnosis of AN, and 161 had a BMI <13 kg/m² as well as a length of stay ≥28 days, with 43 of these (27.3%) discontinuing therapy in the first four weeks. None of these 43 patients had refeeding syndrome. A total of 103 patients with a BMI <13 kg/m², a stay of ≥4 weeks, and available laboratory data per the inclusion criteria were included in this study. These 103 patients had complete data sets, i.e., the laboratory values were available weekly (+− 2 days) for all measurement times. Most of the patients with incomplete data sets were discharged or transferred before the end of the 28 days, mostly due to lack of motivation—in none of these cases was a transfer necessary due to critical deterioration of the general condition. All participants were female and aged 23.8 ± 5.3 (range = 18–47) years. No males in the weight range <13 kg/m² were admitted in the observation period.

The weekly change in weight and BMI is shown in Table 1. The weight of the female patients increased during four weeks by an average of 1.0 ± 0.5 kg/week, and by a total of 4.2 ± 2.0 kg over four weeks (mean, SD). This weight change corresponds to a total increase of 1.6 ± 0.8 BMI units in four weeks (*p* = 0.001). None of the patients experienced a critical deterioration of their physical condition, and all patients improved their performance level with weight gain (sit up, squat, stand (SUSS) test score as specified by the MARSIPAN workgroup (32).

The weight gain was highest in the first week with an average of 1.9 ± 2.0 kg, leveling off to 0.7–0.8 kg/week in the following three weeks. The maximum increase observed in the first week was 8.4 kg, which was mostly due to rehydration (traceable by hematocrit decrease). In 20 patients (19%), the weekly weight gain in the first week was between 0.1 and 0.6 kg, in 13 (13%) between 0.7 and 1.0 kg, in 22 (21%) between 1.1 and 2.0 kg, in 24 (23%) between 2.1 and 4.0 kg, and in 13 (13%) >4.0 kg. In 11 patients (11%) a weight stagnation or weight loss was recorded in the first week, and this group had a mean weight loss of 0.7 kg (max. 2.2 kg to min. 0 kg).

**Figure 1:**
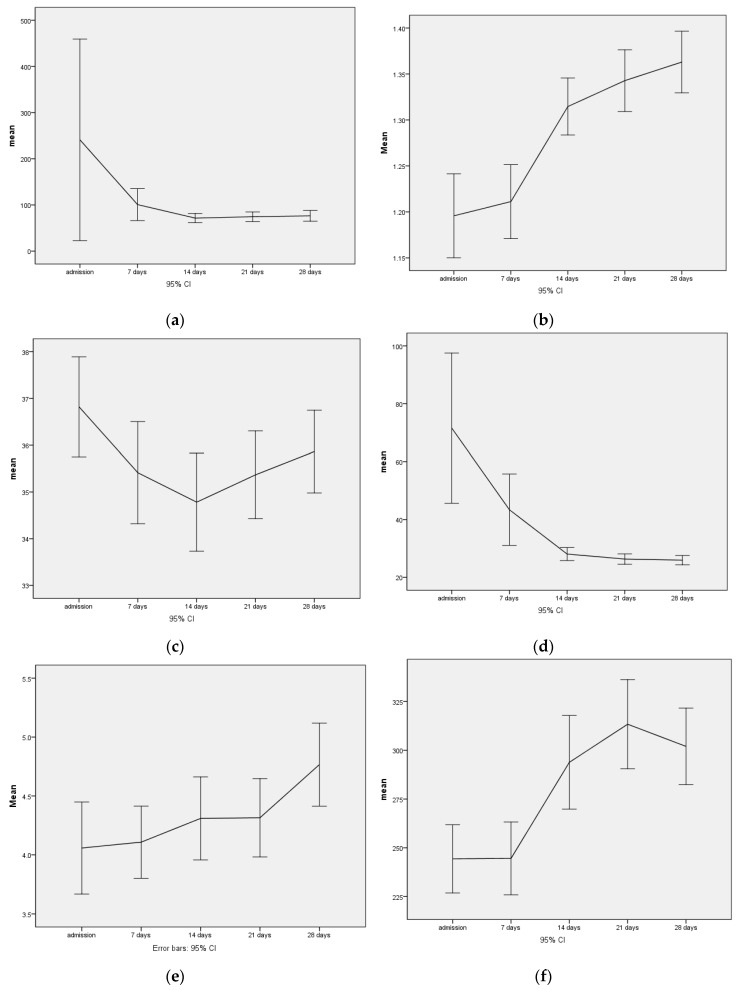
The course of creatine kinase (CK), inorganic phosphate (Phos) × 100, hematocrit (HK), and aspartate aminotransferase (GOT) leukocytes and platelets over four weeks with F-values of the measurement repeatability analysis. T1 to T5 (T1 intake, followed by weekly intervals of the laboratory survey). (**a**) Creatin kinase (F 2,3 df 4, *p* 0.056.); (**b**) Phosphate (F 23,9, df 4, *p* 0.000); (**c**) Hematocrit (F 12,3 df 4, *p* 0.000); (**d**) GOT (F 13,8 df 4, *p* 0.000); (**e**) Leucocytes (F 8,9, df 4, *p* 0.000); (**f**) Platelets (F 19,8, df 4 *p* 0.000.). T1, admission; T5, 28 days after admission.

All patients received 200 mg thiamine daily for the first four weeks and 612–1024 mg phosphate daily for the first two weeks. With this supplementation, serum phosphate levels increased during the observation period (Table 2, Figure 1). On admission, 7 (6.2%) of the 103 subjects had hypophosphatemia with values <0.75 mmol/L, and one subject had a critical value <0.5 mmol/L. After one week, only five patients had slightly decreased serum phosphate levels (<0.75 mmol/L) and after two weeks only three patients.

The decrease in the mean creatine kinase values during the observation period of the first four weeks of treatment clearly shows that the increase in energy intake and the consecutive significant weight gain right at the beginning of treatment was not associated with an increased risk of rhabdomyolysis (Table 2, Figure 1). Strongly elevated values for creatine kinase (>1000 U/L) were only observed in one patient at intake with 9941 U/L, which completely normalized within fourteen days with adequate nutrition and phosphate substitution.

While critical hyponatremia with values <125 mmol/L also occurred only on admission in one patient, the initially already low average value for hematocrit fell from 37% to 35% after fourteen days, and then slowly rose again to 36% after four weeks. Accordingly, up to three (different) patients with their hematocrit fell below the critical value of 25% at the first three measurement points. After four weeks, the values in two patients were still below the critical value. In parallel to the increase in weight, the transaminases, which were strongly elevated at admission, decreased significantly. Further, 54 (51.9%) of the patients had elevated GOT and 62 patients (59.6%) had elevated GPT > 35 U/L at admission, only 12 patients (11.5%) still had elevated values of GOT after four weeks, and the number of pathological values for GPT did not change on average during the four weeks being still *n* = 61 (59.2%).

Similarly, the rapid refeeding led to a highly significant increase in thrombocytes and leukocytes (Figure 1, Table 2), while the hemoglobin, in parallel to the hematocrit, dropped from an initial average of 12.5 ± 1.76 g/dL to 11.5 ± 1.69 g/dL in the third week, and stabilized at 11.6 ± 1.5 g/dL by week 4.

## 4. Discussion

In the sample of 103 adult and severely malnourished patients with AN described here, an oral refeeding protocol with 2000 kcal/d as an initial target and weight-development-adapted increases in energy intake was applied. On average, the patients’ body weight increased by more than 1040 g per week over four weeks and hypophosphatemia could be avoided with phosphate substitution. With this accelerated refeeding protocol, no case of refeeding syndrome (RS) was observed.

### 4.1. Physiological Effects of the Refeeding Protocol in the Present Study

Hyperhydration was common among the patients during the first four weeks of refeeding. This was self-limiting in most cases with only a small number of patients requiring oral diuretics. The initially low average hematocrit value fell significantly within the first fourteen days as a sign of hyperhydration, only to rise slowly thereafter; however, sodium levels remained stable or increased slightly on average, so that the cause of hyperhydration is unlikely to be inadequate vasopressin release or polydipsia. There is no indication that the drop in hematocrit could be avoided with slower refeeding. Hyperhydration could lead to cardiac problems, including myocardial dystrophy that has been associated with malnutrition [14]. In the present sample, the decrease in hematocrit was transient and clinically manageable without cardiac complications. A significant decrease in transaminases was observed as a sign of improvement in general body condition associated with weight gain. The increase in transaminases is considered a sign of the autolysis of body cells associated with the lack of energy [19]. In this respect, the decrease in transaminases can be seen as a sign of improved cellular energy supply. The bone marrow can transform gelatinously [20] when the patient is severely underweight over a prolonged period, thus significantly limiting hematopoiesis. The significant increase in platelets and leukocytes can be seen as a sign of bone marrow recovery and hematopoiesis. On the other hand, hemoglobin did not increase during the observation period, which can be explained by the slower erythropoiesis and the simultaneous hyperhydration with a decrease of hematocrit.

### 4.2. Previously Described Refeeding Protocols and Outcomes in Moderately Malnourished Adolescents with AN

Up till now, initial higher-caloric refeeding was mostly assessed in mildly to moderately malnourished adolescents. For example, in a group of 129 adolescent AN patients (mean intake BMI: 15.9 kg/m^2^), Smith et al. found no case of refeeding syndrome with an initial energy intake of 1585 kcal/d and a rapid increase in energy intake to 3626 kcal/d by day 14 after hospital intake [14]. In that study, phosphate levels were monitored and, if necessary, corrected, which was the case in 75% of the patients. BMI increased by 0.6 BMI units per week over a time of 15 days. Madden et al. [19] also developed a high caloric refeeding protocol for adolescents: in 78 AN patients (78% expected body weight, no BMI information), initial caloric intake started with 2400 kcal/d on days 0–3 by continuous (24 h) nasogastric tube feeding, the weight increased by 2.5 kg in the first week, and by 5.1 kg 2.5 weeks after intake. Using this protocol, none of the patients receiving prophylactic phosphate supplementation developed hypophosphatemia, hypoglycemia, or other signs of RS. In contrast, one of the few case descriptions in the literature of RS refers to a 15-year-old adolescent with a BMI of 11.6 kg/m², low phosphate (0.78 mmol/L), and low glucose (2.6 mmol/L). With phosphate administration, the phosphate level of this patient remained low, and with an energy administration of only 800 kcal/d and a slow increase to 1000 kcal/d after 6 days, an RS developed with severe impairment of the mental state up to delirium [20].

### 4.3. Previously Described Refeeding Protocols and Outcomes in Severely Malnourished, Adults with AN

Gaudiani et al. [9] prescribed 990 kcal/d at the beginning with an increase to 2000 kcal/d after 19 days in 25 adults with AN (mean BMI at admission 13.1 kg/m²), resulting in a BMI increase of 0.5 BMI units per week over a time of 19 days. Hypoglycemia occurred in 44% of patients, 76% showed abnormal liver function, 45% developed hypophosphatemia, and 92% developed hypothermia [9]. The observed complications by Gaudiani may have been due to initial low caloric intake (hypoglycemia and consecutive liver dysfunction) and less proactive phosphate substitution (hypophosphatemia). Hofer et al. [21] reported on 65 adults with AN (mean BMI at intake 13.7 kg/m^2^) who were refed at 437 kcal/d with an initial increase to 1506 kcal/d, leading to an increase of 0.3 BMI units per week over a time of three weeks. This strategy was associated with severe hypokalemia in two patients, but no patient developed hypophosphatemia (due to phosphate supplementation). In 10.5% of patients, the following medical complications occurred: pretibial edema (*n* = 4), organ dysfunction as mild and transient pancreatitis, transient stage 2 renal failure, and urinary tract infection (*n* = 3). In two patients with already existing heart failure at admission with an echocardiographic ejection fraction of 40%, the malnutrition increased the severity of the existing disease. In a recent retrospective, observational study by Matthews et al. with 119 adult AN patients, higher caloric refeeding (group 1: baseline BMI of 17.2 kg/m^2^ and intake of 1500 kcal/d vs. group 2: baseline BMI of 14.9 kg/m² and intake of 1000 kcal/d) along with close medical monitoring stabilized the patients and prevented RS. Our findings are in line with these results, and firstly, extend to an even more malnourished group of patients and second, to higher initial caloric intake.

### 4.4. Relevance of the Findings with Respect to Current Guideline Recommendations

Given these data, it is important to question the recommendations of the current guidelines for the management of AN. The German S3 guideline refers to the U.S.–American studies in adolescents, which recommend faster re-nutrition, but with the remark that at the time of the guideline update no comparable studies from the German-speaking countries and in adults were available. Other guidelines, however, seem to be based on older studies in which the paradigm shift towards faster refeeding had not yet become apparent. For example, the current guidelines for adults with AN (Table 3) recommend a consistently low energy intake, which does not allow for adequate weight gain.

Recent data implies that combined with close medical monitoring, a more accelerated approach appears to be safe, minimize complications, prevent mortality, and to reduce the length of stay in hospitals for patients with AN; however, as long as there is still not sufficient evidence-based data to support a new, general recommendation for severely malnourished, adult patients, nutritional rehabilitation must remain tailored to the individual patient.

There is still a need for systematic research to finally answer the question, which initial refeeding strategy for managing severe malnutrition should be the first-line treatment for AN patients. Further studies are needed that allow a detailed analysis of energy intake, weight change, and any associated complications. The approach presented in the current analysis appears promising and, to our knowledge, unique in this form in adults concerning faster and at the same time safe refeeding.

The strength of the present study is the large sample size and the uniform medical and therapeutic care under the largely standardized medication and diagnostics. One of the methodological limitations is that this is not a randomized patient selection, but a naturalistic setting in a single clinic. A randomized controlled trial (RCT) comparing “slow or regular” versus “fast” refeeding would be the next step to provide evidence for changing refeeding guidelines. We analyzed data from a consecutive cohort of extremely underweight patients. For such patients, inclusion in an RCT might be problematic for ethical reasons. With the chosen procedure, there was no evidence of critical deterioration of the physical condition, on the contrary, a consolidation of critical health status due to the rapid increase in weight was observed. Another limitation of the study is that the individual energy intake of the patients as well as adherence to the dietary recommendations and frequency of contingency measures were not objectively recorded and could only be given with a description of the general refeeding protocol that was as accurate as possible. On the other hand, an exact and objective recording of the energy intake of patients with AN is difficult to achieve, even under controlled conditions, and the comparatively high and rapid initial weight gain proves that correspondingly high energy intake must have taken place. Even though low blood glucose levels and thus hypoglycemia have been reported during refeeding in patients with AN, data on blood glucose levels is not included in the manuscript. To our experience hypoglycemia does not occur if the patients are sufficiently nourished, however, we cannot be sure that there were periods of hypoglycemia in the patient cohort described in our study.

Key messages:

(1) There are few evidence-based high caloric refeeding protocols for severely malnourished adult AN patients.
(2) In the present study, a rapid initial weight restoration strategy targeting oral energy intake of 2000 kcal/d from day one of treatment resulted in an average weight gain of 1040 g/week over four weeks. Combined with close medical monitoring and supplementation of phosphate and thiamine, this accelerated refeeding strategy was not associated with refeeding complications in severely malnourished adult AN patients.
(3) To derive safe and commonly accepted guidelines, further and prospective studies comparing different refeeding regimes are required.
(4) Following previous recommendations [18], the findings of the present study do not constitute medical advice. While the acknowledgment of the accelerated refeeding protocol presented in this study may assist in rethinking models of nutritional management of adult, severely malnourished AN patients, the judgment of the treating professional about the choice of an adequate refeeding strategy for an individual patient should prevail.

## 5. Conclusions

In summary, data from this naturalistic study indicate that the rapid refeeding strategy described here was effective and safe and that RS was not a consequence of more rapid refeeding. Future studies should further assess the efficacy and safety of rapid refeeding protocols in adults with AN who are severely underweight.

## Figures and Tables

**Table 1:** Weekly change in weight and body mass index.

	Weight	BMI		Weight	BMI
Week 1	31.5 ± 3.6	11.5 ± 0.9	Week 1–2	1.3 (0.6/3.0) ***	0.5 (0.2/1.1) ***
	(23.5–40.1)	(9.5–13.0)		(−2.2–8.4)	(−0.8–3.4)
Week 2	33.4 ± 3.7	12.3 ± 1.1	Week 2–3	0.8 ± 0.9 ***	0.3 (0.1/0.5) ***
	(24.0–41.5)	(9.7–15.2)		(−1.2–3.4)	(−0.5–1.4)
Week 3	34.2 ± 3.6	12.6 ± 1.1	Week 3–4	0.7 ± 0.7 ***	0.2 (0.1/0.4) ***
	(26.0–41.5)	(9.8–15.9)		(−0.8–2.8)	(−0.4–1.2)
Week 4	35.0 ± 3.5	12.8 ± 1.1	Week 4–5	0.8 (0.4/1.1) ***	0.3 (0.1/0.5) ***
	(26.5–42.0)	(10.0–16.1)		(−1.4–1.9)	(−0.5–0.8)
Week 5	35.7 ± 3.4	13.1 ± 1.1	Week 1–5	4.0 (2.9 / 5.0) ***	1.4 (1.1/1.9) ***
	(27.8–43.9)	(10.0–16.9)		(−2.5–9.7)	(−0.8–4.1)

***, *p* < 0.001; t-test for normally distributed data are presented as mean (SD), and for non-normally distributed data as median (25/75 percentile) using a Wilcoxon test. change; BMI: body mass index; data in parenthesis are ranges.

**Table 2:** Laboratory values at admission (T1) and after four weeks (T5), Mean and SD for 103 pt.

	CK	Phos	Sodium	GOT	GPT	Leuco	Haematocrit	Hb	Pla
cut off	>170 U/L	<0.75 mmol/L	<125 mmol/L	>35 U/L	>35 U/L	<3.98 G/L	<25 %	<11.2 g/L	<182 G/L
At admission	222 ± 974	1.15 ± 0.29	138.7 ± 4.7	67.9 ± 117.8	85.6 ± 121.9	4.1 ± 1.8	37.0 ± 5.0	12.5 ± 1.8	242 ± 81
	(27–9941)	(0.46–1.92)	(112.0–152.0)	(15.3–1078.0)	(8.3–742.0)	(1.1–14.0)	(22.9–44.7)	(7.5–15.6)	(45–494)
After four weeks	75 ± 54 ***	1.34 ± 0.18 ***	141.3 ± 2.7 ***	25.9 ± 8.0 ***	42.3 ± 21.6 ***	4.8 ± 1.8 ***	36.1 4.2 **	11.6 ± 1.5 ***	300 ± 92 ***
	(22–374)	(0.94–1.88)	(133.0–148.0)	(12.7–53.0)	(10.7–133.0)	(1.6–12.8)	(23.0–42.0)	(6.6–14.3)	(185–849)
pathologic at admission	19 (103)	7 (103)	7 (103)	54 (103)	62 (103)	54 (103)	23 (103)	21 (103)	21 (103)
	18.4%	6.8%	6.8%	52.4%	60.2%	52.4%	22.3%	20.4%	(20.4%)
pathologic after four weeks	6 (103)	0 (103)	0 (103)	12 (103)	61 (103)	37 (101)	22 (101)	36 (101)	0 (101)
	5.8% *	0% *	0% *	11.7% ***	59.2%	36.6% *	21.8%	35.6% *	(0%) ***

The T-test was used for normally distributed differences in blood values between two measurement points. A Wilcoxon test was used for non-normally distributed differences. For the percentage change in the proportion of pathological blood values, the two-sided Fischer’s exact test was used * *p* < 0.05; ** *p* < 0.01;***. *p* < 0.001; data in parenthesis are ranges. Abbreviations: CK: creatine kinase; Phos: inorganic phosphate; GOT: aspartate aminotransferase; GPT: alanine aminotransferase; Leuco: leukocytes; Hb: hemoglobin; Pla: platelets; SD, standard deviation.

**Table 3:** Current, national refeeding guidelines for malnourished patients with anorexia nervosa.

Guidelines	Age	Recommended Energy Intake
Australia and New Zealand	adult	1400 kcal/d [22]
Europe [23]	adult	start at 10, slowly increase to 15 kcal/kg/d (Day 1–3)
United Kingdom: Royal College of Psychiatrists [24]	adult	10–20 kcal/kg/d
United Kingdom: MARSIPAN [25]	adult	5–20 kcal/kg/d
American Psychiatric Association/American Dietetic Association [26]	adult	30–40 kcal/kg/d (1000–1600 kcal/d)
United Kingdom: Junior MARSIPAN [27]	<18 years	20 kcal/kg/d
